# Inducing Psychedelic-Like States Through Brain Stimulation: A Review of Mechanisms, Clinical Evidence, and Psychotherapeutic Implications

**DOI:** 10.1192/bjo.2026.11217

**Published:** 2026-06-30

**Authors:** Sunandan Banerjee

**Affiliations:** Black Country Healthcare NHS Foundation Trust, Birmingham, United Kingdom

## Abstract

**Aims:**

To examine whether psychedelic-like states of consciousness can be induced through brain stimulation techniques, evaluate their potential advantages over pharmacological psychedelics, and explore how stimulation-assisted psychotherapy may offer a clinically viable model for mental health treatment.

**Methods:**

A structured narrative review was conducted using PubMed and PsycINFO,examining peer-reviewed studies of non-invasive and invasive brain stimulation techniques, including transcranial magnetic stimulation (TMS), transcranial alternating current stimulation (tACS), transcranial direct current stimulation (tDCS), and deep brain stimulation (DBS). Literature describing subjective phenomenology, neuroimaging findings, and therapeutic outcomes was reviewed alongside evidence from psychedelic-assisted psychotherapy to enable mechanistic comparison. Findings were synthesised thematically with reference to clinical relevance.

**Results:**

Across multiple studies, targeted brain stimulation was shown to induce transient alterations in perception, emotional salience, self-experience, and cognitive flexibility–phenomenological features overlapping with psychedelic states. Neuroimaging and electrophysiological data indicate that both psychedelic states and brain stimulation modulate large-scale brain networks, particularly through reduced dominance of the default mode network, increased global connectivity, and altered thalamocortical and corticolimbic signalling.

Unlike pharmacological psychedelics, brain stimulation avoids systemic drug exposure, reducing risks of prolonged perceptual disturbance, pharmacokinetic unpredictability, substance interactions, and psychosis precipitation related to serotonergic agonism. Stimulation parameters can be titrated, paused, or terminated in real time, offering enhanced safety, reproducibility, and clinical governance.

Importantly, evidence from psychedelic research indicates that therapeutic benefit is primarily mediated through psychotherapeutic processes–such as insight generation, emotional processing, and narrative restructuring–rather than the altered state alone. Brain stimulation may similarly act as a catalyst for psychological change by transiently increasing neural and cognitive flexibility, thereby enhancing responsiveness to psychotherapy across conditions including depression, trauma-related disorders, and addiction.

**Conclusion:**

Brain stimulation techniques may offer a controllable, non-pharmacological means of accessing key neural and psychological mechanisms associated with psychedelic states, while mitigating many drug-related risks. When integrated with structured psychotherapy, stimulation-assisted models may provide a pragmatic translational pathway for harnessing psychedelic-relevant mechanisms within existing mental health services. Further research is required to establish optimal stimulation parameters, safety profiles, and disorder-specific applications before routine clinical use.

